# CHDmap: One Step Further Toward Integrating Medicine-Based Evidence Into Practice

**DOI:** 10.2196/52343

**Published:** 2024-04-19

**Authors:** Jef Van den Eynde

**Affiliations:** 1 Department of Cardiovascular Sciences, KU Leuven, Leuven, Belgium

**Keywords:** artificial intelligence, clinical practice, congenital heart disease, decision-making, evidence-based medicine, machine learning, medicine-based evidence, patient similarity networks, precision medicine, randomized controlled trials

## Abstract

Evidence-based medicine, rooted in randomized controlled trials, offers treatment estimates for the average patient but struggles to guide individualized care. This challenge is amplified in complex conditions like congenital heart disease due to disease variability and limited trial applicability. To address this, medicine-based evidence was proposed to synthesize information for personalized care. A recent article introduced a patient similarity network, CHDmap, which represents a promising technical rendition of the medicine-based evidence concept. Leveraging comprehensive clinical and echocardiographic data, CHDmap creates an interactive patient map representing individuals with similar attributes. Using a k-nearest neighbor algorithm, CHDmap interactively identifies closely resembling patient groups based on specific characteristics. These approximate matches form the foundation for predictive analyses, including outcomes like hospital length of stay and complications. A key finding is the tool’s dual capacity: not only did it corroborate clinical intuition in many scenarios, but in specific instances, it prompted a reevaluation of cases, culminating in an enhancement of overall performance across various classification tasks. While an important first step, future versions of CHDmap may aim to expand mapping complexity, increase data granularity, consider long-term outcomes, allow for treatment comparisons, and implement artificial intelligence–driven weighting of various input variables. Successful implementation of CHDmap and similar tools will require training for practitioners, robust data infrastructure, and interdisciplinary collaboration. Patient similarity networks may become valuable in multidisciplinary discussions, complementing clinicians’ expertise. The symbiotic approach bridges evidence, experience, and real-life care, enabling iterative learning for future physicians.

Evidence-based medicine (EBM), built on the foundations of randomized controlled trials (RCTs), is good at providing average estimates for treatments or outcomes in the average patient. While EBM has resulted in important clinical guidelines, it does not solve the real clinical quandaries: patients appear for care individually, each may differ in important ways from an RCT cohort, and the physician will wonder each time if following EBM will provide best guidance for this unique patient. This is particularly the case for complex and heterogeneous populations, such as those with congenital heart disease (CHD). Indeed, in congenital cardiology, RCTs are both difficult to conduct and commonly not definitive. The complexity of disease, clinical heterogeneity within lesions, and the small number of patients with specific forms of CHD severely degrade the precision and value of estimates of average treatment effects in the average patient provided by RCTs.

In response to mounting concern about the value of EBM for decision-making, we have previously proposed medicine-based evidence (MBE) as a means of synthesizing all available information and applying it to the individual patient [1]. Briefly, we proposed that whenever a physician needs to decide a patient’s treatment plan, a library of patient profiles would be interrogated. A nearest neighbor algorithm would then find “approximate matches,” a group of patients who share the greatest similarity with the index case. Some of these matches would and others would not have received a certain treatment or developed a certain outcome, such that specific analyses tailored to the clinical question could be performed within this pool of approximate matches. We envisioned that this approach would represent a major step toward true personalized medicine, as individualization of treatment would shift from today’s intrinsically subjective human-driven assessment toward a more objective, data- and model-driven process that is more descriptive, integrative, and predictive.

In their recent article, Li et al [2] introduced CHDmap, an innovative patient similarity network (PSN) designed to prognosticate outcomes among patients with CHD. By leveraging comprehensive clinical and echocardiographic data sets from 4774 surgical cases, the PSN manifests as an interactive, zoomable electronic cartography, wherein each node symbolizes an individual patient, and internode distances delineate their similarity. This user-centric software empowers practitioners to delineate specific patient attributes—such as age, gender, CHD classification, and echocardiographic metrics—tailoring the analysis to the case at hand. The program subsequently uses a k-nearest neighbor algorithm to identify a cohort of closely resembling peers according to the top-k parameter or similarity threshold. This assemblage of approximate matches serves as the foundation for diverse predictive analyses, encompassing variables like hospital length of stay, complications, and survival. This way, CHDmap allows for conducting real-time clinical trials that are specifically tailored to the individual patient, based on historical cases with a similar clinical profile. A key finding from the study by Li et al [2] was the tool’s dual capacity: not only did it corroborate clinical intuition in many scenarios, but in specific instances, it prompted a reevaluation of cases, culminating in an enhancement of overall performance across various classification tasks.

The tool has been made publicly available [3] and represents a promising technical rendition of the MBE concept. According to the authors, future generations of the software will be uploaded in time, further expanding the possibilities of CHDmap, including the following: (1) Labeling and visualization of increasingly complex and rare CHD types—currently, only some major subtypes (atrial septal defect, patent foramen ovale, ventricular septal defect, patent ductus arteriosus) are depicted in the map overview; as the underlying data set expands, patients with more complex anatomy may be visualized as well. (2) More granularity in data—in a similar manner, the width of the underlying data set (ie, number of cases) and its depth (ie, number of variables) will likely increase, allowing for more precise matching and examination of more aspects of decision-making. (3) Long-term outcomes—currently, only in-hospital outcomes can be considered within CHDmap, but future generations of the software may allow for long-term outcomes to be analyzed. (4) Comparisons of specific treatment options—once in-depth data on various treatments become available, the optimal treatment for an individual patient may be examined through real-time clinical trials within CHDmap, where outcomes after initiation of various treatments are compared among a group of approximate matches. (5) Artificial intelligence–driven weighting of indicators—the default setting in CHDmap allocates to each indicator the same weighting, whereas physicians can modify these weights based on their prior knowledge; the latter option allows accounting for the fact that weights are likely to differ depending on the clinical setting and the question at hand. With future generations of CHDmap, the authors may implement an artificial intelligence model to dynamically allocate weights to each of the indicators.

CHDmap undeniably signifies a significant stride toward the actualization of MBE. Just as with any statistical methodology, the principles of implementation science will play a pivotal role in optimizing the widespread integration of this tool into clinical practice [4]. Medical practitioners will need to be trained to use these tools correctly and to ensure they are aware of the perks and pitfalls of the PSN (eg, knowing that there is a trade-off between increasing similarity and increasing statistical power or being able to correctly interpret the certainty associated with a specific prediction). Data infrastructure will need to be in place, and continued efforts should be made to establish multicenter clinical registries with in-depth and up-to-date information collection. Furthermore, collaboration between health care professionals and experts in data science will be required to ensure these novel technologies can benefit our patients, taking into account issues regarding data quality and privacy.

Finally, at some point in the future, tools like CHDmap may become routinely used to support team discussions. Rather than replacing the clinician, they should be embraced as assistive technology enhancing overall clinical efficacy. This symbiotic approach serves to harmonize real-life patient care with prior experience and established evidence. This way, we can truly start to achieve the incremental benefits of future generations of physicians learning from previous ones.

## Abbreviations

CHD: congenital heart disease
EBM: evidence-based medicine
MBE: medicine-based evidence
PSN: patient similarity network
RCT: randomized controlled trial
